# Collaborative Policymaking: a qualitative systematic review of advice for policymakers

**DOI:** 10.12688/openreseurope.18440.1

**Published:** 2024-09-18

**Authors:** Paul Cairney, Claire Toomey

**Affiliations:** 1 Faculty of Arts and Humanities, University of Stirling Division of History Heritage and Politics, Stirling, Scotland, FK94LA, UK

**Keywords:** Collaborative policymaking, collaborative governance, collaboration, policy, policymaking

## Abstract

**Background:**

Complex policy problems are not amenable to simple solutions by a few powerful policy actors in one central government. They require collaboration across government and between actors inside and outside of government. However, this *requirement* for collaboration is no guarantee of collective action. Further, it is difficult to know how to collaborate effectively. We searched the academic and grey literature for advice on how to foster collaborative policymaking.

**Methods:**

We conducted a qualitative systematic review (2024) of peer reviewed journal articles (Web of Science) and grey literature reports (Policy Commons). Each article or report had to inform advice on collaborative policymaking. We used an immersive and inductive approach to identify key themes and relate the results to well-established insights from policy theories.

**Results:**

86 texts meet the inclusion criteria (49 Web of Science, 37 Policy Commons). Most provide broad definitions of collaborative policymaking, which are similar to definitions of collaborative governance (and connected aims such as policy co-creation). Many assert or assume that greater collaboration, across and inside/outside of government, will improve policymaking and policy. Few individual studies give advice on how to collaborate effectively, but they combine to identify common features of collaboration.

**Conclusions:**

We synthesise the available advice to identify five main features of collaborative policymaking: plan and prepare to collaborate, such as by designing rules and allocating resources; create a sense of collective purpose, such as by setting a boundary around the collaboration and co-producing a common vision; foster creative methods to visualise collaboration and design policy; create new forums to supplement formal collaboration; and clarify the roles and skills essential to each collaborative task.

## Plain language summary

Policy problems are complex and not amenable to simple solutions. Gone are the old stories of elite analysts showing a powerful central government how to solve problems. In their place are new stories of the need for many organisations to collaborate to get things done. Collaboration is necessary across government departments, and levels of government, because problems transcend traditional sectoral and jurisdictional boundaries. Collaboration with citizens, stakeholders, and organisations outside of government is essential, to reflect the need to widen participation, gather information, and boost support for policy change.

However, this *requirement* for collaboration is no guarantee of action, and there is a big gap between requirement and reality. It is also difficult to know how to get started or to collaborate effectively.

Our aim was to find practical advice on how to design or support collaborative policymaking. We searched for lessons not only from academic articles but also governments, think tanks, and other organisations sharing transferable lessons of successful collaboration.

This search for practical lessons proved to be like the search for a needle in a haystack. Most studies assert the importance of collaborative policymaking, but few define it well, and fewer describe how to do it.

That said, we find enough information to identify five main practices associated with effective collaboration. Most effective action involves planning and preparation, to help create a well-understood sense of collective purpose, produce a common vision with partners, use creative methods to visualise collaboration, create new informal forums to support formal collaboration, and ensure that enough people have the specialist skills and resources to fulfil each essential task. These studies reject the idea that we can produce a detailed blueprint. Rather, broad principles help people and organisations to work together to turn general aims into concrete action appropriate to their collaboration.

## Introduction

We present a qualitative systematic review of advice for collaborative policymaking. The context for this review is high demand for usable advice from academic research and practitioner experience. State-of-the-art academic research rejects an old story of elite policy analysis and centralised authority in favour of a new story in which collaborative policymaking is increasingly necessary to effective government ( Cairney & Toomey, 2024). A common theme in multiple policy concepts and theories is that ‘complex policy problems do not respect traditional government boundaries, and require collaborative responses across government and between governmental and non-governmental actors’ ( Cairney, 2024). However, while many scholars agree with the vague proposition that collaborative policymaking is essential, they rarely identify what exactly it is and how to do it. Similarly, there is high demand within governments to learn how to define and foster collaborative policymaking. Indeed, a key impetus for our review was heightened demand in the European Commission Joint Research Centre (JRC) to learn, from state-of-the-art policy and practice, about how exactly to foster effective collaboration ( Cairney & Toomey, 2024).

In that context, our initial aim was to find evidence-informed advice – on the meaning and practice of collaborative policymaking - that is usable in the real world. In political systems, usability requires both political and technical feasibility: an initiative or solution needs to be acceptable to enough powerful actors to initiate the work, and work as intended if implemented. For example, is not politically feasible for the JRC to recommend a complete systemic redesign in the European Commission, but there is a high focus on innovation at the start of a Commission’s new term, and scope for small scale and implementable changes to produce quick wins that encourage further change (2024: 4–5). For example, if it is essential for many colleagues, spread across Directorates General (DGs), to collaborate more effectively with each other and their stakeholders, what insights could inform immediate changes to individual, organisational, and procedural cultures and practices?

This real-world demand for actionable insights informs our search for lessons, not only in academic but also grey literatures. Usually, a systematic review draws primarily or exclusively from peer reviewed academic journal articles, using multiple databases to get closer to comprehensive coverage. However, this initial stipulation rules out the possibility of collecting usable insights from high quality and relevant sources in practice-based research. The latter could offer qualitative and actionable research from actors who appreciate the role of policymaking context and the limits to delivering too abstract ‘best practice’ approaches. Therefore, we draw on a search in the academic (Web of Science) and grey literature (Policy Commons) to compare insights from different sources. Then, based on our engagement with these databases, we deduced that a process of snowballing to identify the foundational sources for insights (and connections to related concepts and initiatives) was a better use of scarce resources than the same search in multiple databases (see Methods).

This real-world demand for usable advice from scholars and practitioners also informs how we report the results. To make full sense of the advice, we must relate it to the two main reference points that scholars and practitioners use: how people want policymaking to work, and how it really works. In other words, people relate advice to aspirational and empirical stories. Governments tell aspirational stories to convey what they need to do and look like they are doing. For example, they may present an image of the *policy cycle* to describe a linear and orderly policy process consisting of essential policymaking functions, and use slogans such as *evidence-based policymaking* to exemplify such aspirations ( Cairney, 2016). Academics also tell aspirational stories to connect collaborative policymaking to the pursuit of better democracy, in which meaningful cooperation between policymakers, citizens, and stakeholders can legitimise and inform policymaking ( Ansell & Gash, 2008). However, policy theory-informed empirical studies tend to tell a less optimistic story of how policy processes really work, highlighting policy and policymaking complexity and the constraints as well as facilitators of change.

Both reference points inform the meaning of any new advice on collaborative policymaking. For example, studies may begin by stating what we want or require from policymaking: complex policy problems require high levels of meaningful collaboration by actors spread across multiple government departments, levels of government, and/ or public sector organisations, as well as with networks of nongovernmental actors (NGOs) who influence the substance and delivery of policy ( Cairney, 2024). However, they may also suggest that complex policymaking systems do not necessarily meet these requirements or provide conditions conducive to collaboration. In that context, while there is limited value in relating advice only to a policy process that could not exist, we also avoid the unhelpful conclusion that positive change is completely out of governmental control. We balance the ambition to get closer to aspirational models of policymaking with the acceptance of systemic limits to action. This approach prompts a search for pragmatic advice that offers hope for individual and collective responses but tempered by engagement in a system over which they do not have full understanding or control. What advice do scholars and practitioners offer, and how can we relate it to audiences such as the European Commission? To that end, we cast our net as widely as possible, to search for any potentially relevant advice, including aspirational advice on what good collaborative policymaking might look like, as well as pragmatic and context-specific accounts of what worked, for whom, and in what context. If a study used the phrase ‘collaborative policymaking’, and offered or informed advice, we reviewed its finding.

We summarise general advice (Results) and synthesise this advice to identify five relevant themes (Discussion). There is no uniform blueprint or ‘how to do it’ guide’. Rather, there are common practices associated with positive accounts, including the value of planning and preparation, creating a sense of collective purpose, fostering creative methods to visualise collaboration, creating new forums to supplement formal collaboration, and ensuring capacity by identifying the roles and skills essential to each collaborative task. We conclude by noting that, while these themes may seem frustratingly vague, an essential part of collaborative policymaking is to work together to make sense of essential aims and practices in specific contexts. In other words, the insistence of hard and fast rules for collaboration may undermine collaborative policymaking. If so, the search for a uniform how-to-guide for collaborative policymaking is a fool’s errand.

## Methods

We incorporate ‘limitations’ in this section because they inform strongly our discussion of methods. We begin by adapting Kuckertz and Block’s (2021) guidance on describing systematic reviews, then describe our response to key limitations.

*Rationale*. We seek to understand advice on how to design, support, or improve the effectiveness of collaborative policymaking. Our review relates to the European Commission pursuit of more effective collaboration (for example, the JRC commissioned seven working days to support Cairney’s role in analysis). It also represents the first of a series of reviews for the Horizon Europe funded project *Healthy Working environments for all Ages: An evidence-driven framework* (WAge). WAge’s Work Package 1 seeks to support a process of collaborative policymaking to foster the uptake of usable evidence, which requires us to understand how collaborative policy processes or initiatives work.

*Engagement with previous reviews.* Cairney leads a wider team conducting a series of qualitative systematic reviews that connect insights on key aims or strategies to policy theory insights ( Cairney *et al.*, 2021; Cairney & Kippin, 2022; Cairney *et al.*, 2023). This is the fourth review submitted to Open Research Europe, but it diverges from the first three in relation to focus (the first set focused on equity) and sources (the first set did not explore the grey literature). Previous reviews of research on collaboration are included in our dataset (e.g. Ansell & Gash, 2008).

*Research/guiding questions.* Our most general guiding question was: What advice do scholars and practitioners offer on collaborative policymaking? We used the following sub-questions to guide our initial search and reflection:

1. How many studies use the term ‘collaborative policymaking’ to describe their object of study?
2. How do these studies describe collaborative policymaking? For example, do authors describe their pursuit of collaboration and how to do it, or do they study efforts in government to encourage collaboration?
3. What factors do they describe as constraining or facilitating collaborative policymaking?
4. In what context does this pursuit of collaborative policymaking take place? For example, within one government or across multiple levels? Across policy sectors? Collaboration between government and non-governmental actors?
5. What findings or recommendations do these studies provide?
6. To what extent are these lessons transferable to other contexts?

We anticipated that each included text would define collaborative policymaking then offer at least one of three kinds of advice:

1. Aspirational: what would good collaborative policymaking look like?
2. General: what context-free advice do people give to foster collaboration?
3. Context-specific: what works, for whom, and in what context, and what lessons are transferable to other contexts? While the wording is resonant with realist review, we did not expect to find enough information to conduct the review in that way (based on our previous team experiences).

*Databases and initial search terms.* We began by searching for advice from one main database of peer reviewed academic texts (Web of Science, WoS) and of the grey literature, including government, non-governmental organisation, academic and think tank reports (Policy Commons, PC). We used the search term ‘collaborative policymaking’, anticipating that it would (1) maximise coverage among texts using this term and require a high level of manual searching (which was inevitable, given our need to search manually for advice), and (2) provide only partial coverage of relevant work using different terminology. We address the latter by snowballing some strongly connected terms (e.g. ‘collaborative governance’) and conducting separate reviews for others (e.g. ‘systems leadership’ in progress; see also Aoki *et al.*, 2024 on joined-up and whole of government approaches). We learned from previous reviews that the addition of multiple academic databases had rapidly decreasing returns, and therefore mixed success in relation to the resources involved (e.g. compared to snowballing). Our initial experience with WoS and PC (as well as Overton for further grey literature) suggested that further broad searches would be a poor use of resources.

*Timeliness.* We ran these searches up to 22.5.24 (WoS) and 30.5.24 (PC).

*Manual searches and choices regarding initial inclusion.* We used similar criteria for inclusion as our other three reviews, including publication in English and not restricting initial inclusion in terms of the research method proxy for quality. Our preference was to set an initially low inclusion bar to be able to immerse ourselves in the literature, identify the relevance of texts, and generate a broad narrative of the field to help to make sense of any advice. We modified our previous searches to incorporate the low likelihood that grey literature publications would have the same reference points as academic research (previous reviews required a relevant citation to indicate engagement with policy concepts). As such, the lengthy discussion of mainstream and non-mainstream policy studies – in Cairney *et al.* (2023: 5–6) - is less relevant to this review.

**Figure 1.**
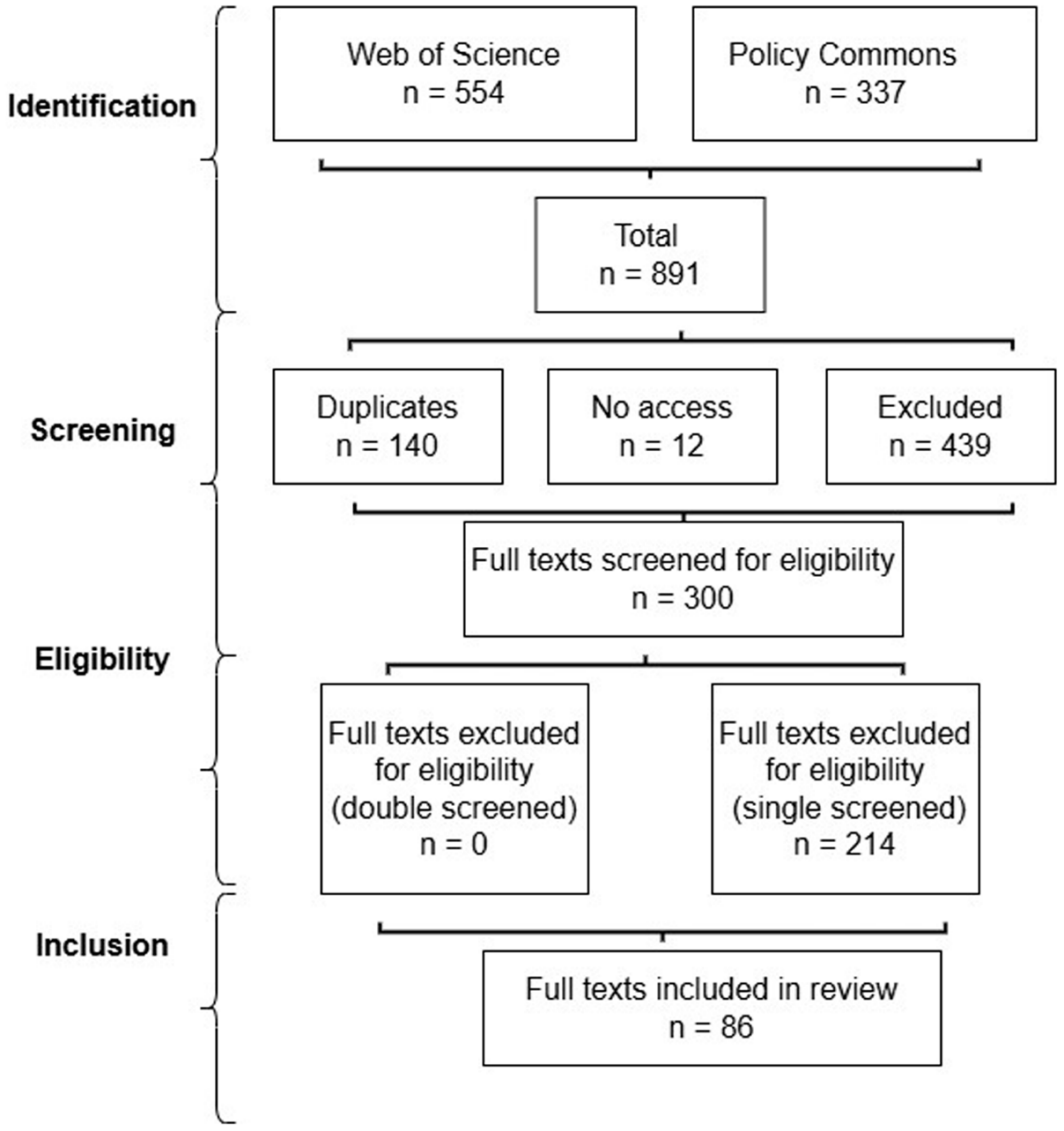
Review process flow chart.

Toomey conducted a manual search of each text in a two-stage process to include texts that: (1) referred specifically to the phrase ‘collaborative policymaking’ in the title, abstract, and/ or introduction, and (2) offered advice in the main text (or information to support advice, such as if describing the effectiveness of collaboration strategies). Toomey found that a remarkably small proportion of texts met both criteria. Even at this stage, the search for advice was akin to seeking a needle-in-a-haystack ( Table 1 and Figure 1).

**Table 1.** Search results 2024.

Database	Search results	Duplicates	No access	Excluded	Included
Web of Science	554	56	5	444	49
Policy Commons	337	84	7	209	37
Grand Total	891	140	12	653	86

*Routine data collection.* Toomey coded the following aspects of each text (in Excel):

- Country/region of study. Publication in English tends to skew the results further to authors in Global North countries focusing on that country. Of the 49 WoS articles: 29% (14) study the US; 10% (5) UK; 8% (4) Netherlands; 6% (3) Sweden; 4% (2) Norway, Italy, Germany; 2% (1) Indonesia, Spain; Singapore; Latin America; Canada, China, Switzerland. Further, 20% (10) had authors across multiple regions including the US, Denmark, Australia, Sweden, Netherlands, Germany, South Africa, Switzerland; Finland, France, Italy, Thailand, Singapore, Columbia, China, Iran, Ethiopia, Pakistan, India (one study compared Argentina, Bolivia, Chile, Columbia, Guatemala, Mexico). The 37 PC are more difficult to categorise since organisation may be more relevant than author country, particularly in the case of European Commission publications (43%, 16). Other include 19% (7) US, 11% (4) Canada, 5% (2) Singapore, Germany; 3% (1) UK, France, Australia, Portugal, Netherlands, Puerto Rico.
- Sectors or issues. Of the 49 WoS articles, most could be categorised broadly as focusing on politics and public administration. When using case studies, environmental issues (including climate change and ecology) were the most discussed (40%, 20), while other topics included health/life science, tourism, and the economy. PC texts exhibit the same pattern of focusing on policymaking and administration generally with, for example, 11% (4) also focusing on environmental issues, plus one-off topics including resilience, finance, trade, and technology.
- Methods or approaches. Of the 49 WoS articles: 61% (30) used qualitative case studies, 22% (11) mixed methods, 8% (4) quantitative, 4% (2) literature reviews. Of the 37 PC: 24% (9) did not specify method, 24% (9) used workshops, focus groups and discussion forums, 22%, (8) were Reviews/Commentary pieces on policymaking issues, while research summaries, data-tools for policymakers, mixed methods were each 8% (3), and 3% (1) case study. Some developed action plans and toolkits but without specifying any method.
- Definitions. We gathered basic information on the extent to which each study defined collaborative policymaking, which we categorised in relation to sub-focus. Of the 49 WoS articles: 22% (11) provided no clear definition, 24% (12) related it to collaborative governance, 18% (9) to partnerships/coalitions, and 4% (2) to policy co-creation. Of the 37 PC texts: 78% (29) provided no clear definition, 8% (3) related it to policy co-creation, 8% (3) to partnerships/coalitions, and 5% (2) to collaborative governance.
- Specificity of advice. We exclude if texts clearly offer no advice, but also note that only a small proportion of included texts provide actionable advice (the rest is information that we can use to generate advice). We categorise the 49 WoS articles as providing: general advice (37%, 18), advice on organisations or implementation (35%, 17), tips or practical advice on collaboration (16%, 8), policy or science-policy nexus advice (10%, 5) or advice on leadership (2%, 1). We categories the 37 PC as providing: general advice (32%, 12), advice on organisations or implementation (16%, 6), policy or science-policy nexus advice (14%, 5), advice on knowledge exchange (14%, 5), advice on technology for collaboration (8%, 3) or advice on leadership (5%, 2).

*Data analysis, aggregation, and presentation.* As with previous reviews, Cairney used an inductive qualitative approach to analyse each text, generate themes manually (Results), and synthesise advice from the results (Results and Discussion). As each previous review notes, ‘the rules associated with this method are less prescriptive than with its quantitative equivalent, suggesting that we (a) describe each key judgement, and (b) foster respect for each authors methods and aims’ (Cairney and Kippin, citing Sandelowski & Barroso, 2007: xv). We did not perform any quantitative tests to assess risk of bias. We present a narrative systematic review (rather than qualitative coding aided by tests for inter-coder reliability).

*Limitations relevant to data gathering and analysis*

The nature of our task *and limitations to our search* influence our approach to data analysis and presentation. We identify five limitations that should inform any interpretation of this review. First, the term *collaborative policymaking* is frequently used too loosely to be useful. In our first team review ( Cairney *et al.*, 2021), the focus on a specific strategy – Health in All Policies – allowed us to identify texts with a clear reference point. In this review, we found that most texts described collaborative policymaking without clarifying its meaning.

Second, this vagueness and loose usage ensures a disparate literature that may focus on different things under the same banner. For example, our initial JRC audience sought to understand the coordination of policymaking across government departments ( Cairney & Toomey, 2024), while others study cooperation across levels of government, or seek to capture activity inside and outside of government:

‘ *Collaborative policymaking* is a generic term that refers to the engagement of nongovernmental actors in decision making relating to some part of the policy process and is inclusive of different forms of participatory policymaking including stakeholder partnerships, advisory committees, public hearings, and negotiated rulemaking’ ( Siddiki & Goel, 2017: 255)

Studies of collaboration inside/outside government often prefer the term *collaborative governance*, which is also defined widely:

‘The processes and structures of public policy decision making and management that engage people constructively across the boundaries of public agencies, levels of government, and/or the public, private and civic spheres in order to carry out a public purpose that could not otherwise be accomplished. ( Emerson *et al.*, 2012: 2, cited in Kim & Siddiki, 2018: 160)
‘A governing arrangement where one or more public agencies directly engage non-state stakeholders in a collective decision-making process that is formal, consensus-oriented, and deliberative and that aims to make or implement public policy or manage public programs or assets. This definition stresses six important criteria: (1) the forum is initiated by public agencies or institutions, (2) participants in the forum include nonstate actors, (3) participants engage directly in decision making and are not merely ‘‘consulted’’ by public agencies, (4) the forum is formally organized and meets collectively, (5) the forum aims to make decisions by consensus (even if consensus is not achieved in practice), and (6) the focus of collaboration is on public policy or public management.’ ( Ansell & Gash, 2008: 544).

Third, we find that no included academic research focuses on giving answers to the practical questions we ask (as expected, it is rarely the aim of academics to provide how-to advice). Most provide a broad and aspirational account of the potential benefits of collaboration, and some attend to the idea of institutional design, but with no real focus on how to collaborate. Fourth, our search for ‘collaborative policymaking’ in the grey literature produced a dispiritingly poor ratio of effort to reward. Searches pick up a remarkably wide range of activity and a loose reference to collaboration, which undermines a targeted or comprehensive search (not as expected, since we anticipated more advice from reports to or from governments). Fifth, much included research identifies what we don’t know, such as the limited extent to which we can track the impact of collaboration from formulation to implementation ( Hoornbeek *et al.*, 2013) or if participants know when collaboration is meaningful or productive ( Vandenbussche *et al.*, 2020).

Overall, the available research – in key academic and grey literature databases - is not well matched to our task and the relevance of its findings are open to wider than usual interpretation. These studies identify the general benefits of wider collaborative policymaking in a liberal democracy, including to gather better information, maintain wide ownership of policy, and foster democratic principles, but they rarely inform directly the design of everyday activities.

We respond in three pragmatic ways. First, during stage 1 of the qualitative systematic review, we kept the bar for inclusion low, to immerse ourselves in the field and search for as many links to relevant studies as possible. Our WoS search produces research on topics such as cross-departmental cooperation, collaborative governance including governmental and non-governmental actors, networks of policymakers and researchers, collaboration in or across multiple levels of government, and complex networks of collaboration focused on cross-cutting policy problems. Our PC search also included topics such as collaboration with business groups and experts, the co-creation of policy with stakeholders and citizens, and considerable work published by the European Commission (especially the JRC). Second, we snowball to identify and learn from key reference points for this research (e.g. Ansell & Gash (2008) and the institutional analysis and development framework ( Ostrom, 2007) are common reference points). Third, we present our synthesis of insights from academic and grey literatures to present our interpretation of practical advice.

More research would be required to do full justice to a disparate field, such as to perform multiple keyword searches to describe each aspect of collaboration across government (including ‘holistic’ or ‘joined-up’ government, approaches to collaboration in fields such as policy design, or terms such as systems thinking or systems leadership).

The complete search protocol, PRISMA checklist, and bibliography of the included texts is stored on the Open Science Framework (OSF) ( https://osf.io/9bk6a/) 

## Results

### The context for advice on collaborative policymaking: informing aspirational and empirical stories

Our advice on collaboration relates to two overarching reference points: how people want policymaking to work, and how it really works. The normative focus has different variants, such as to promote rationalist aims and/ or foster democratic principles. The ‘real world’ focus is informed by multiple empirical policy theories. Both overarching reference points contribute to the same initial three-part story of the imperative to collaborate, as follows.

***Complex policy problems are difficult to define and contain within traditional government boundaries***

Policy problems are not amendable to technical solutions designed by experts at the heart of government ( Cairney, 2021). There is always contestation among many policy actors to define the size, urgency, and cause of policy problems, and therefore who should be responsible for solving them. For example, a government-led response is not always the preference of governments or key stakeholders (many prefer individual responsibility and market solutions over state intervention). Further, if we establish government responsibility, there is contestation to determine which level or sector should take the lead. There is also genuine uncertainty about how to relate key functions (such as problem definition and policy formulation) to wider policymaking principles, such as to ensure that the process is transparent and accountable to citizens, future oriented and preventive, decentralised, built on co-production with stakeholders, coherent, evidence-informed, and with fair procedures and equitable outcomes ( Cairney, 2023). The overall message is that the same policy problem may require involvement by multiple actors across governments, but with the expectation that those actors would define and address the problem in different ways.

***There is no single centre of government to coordinate policymaking***

There is no prospect of processing policy as if by a single brain ( Lindblom, 1959). This level of ‘comprehensive rationality’ would require of one actor the ability to: gather and process all information relevant to policy problems and the likely effect of solutions; produce a rank-ordered and consistent set of government policy preferences; separate policymaker values informing policy aims from the facts necessary to inform and carry out such aims; and, make unilateral choices to maximise the benefits of policy to society without creating winners and losers ( Cairney, 2020: 57–60). Rather, policymakers deal with their ‘bounded rationality’ ( Simon, 1976) by relying on cognitive shortcuts to make quick-enough decisions: strategic methods to clarify aims and seek trusted sources of information, coupled with reliance on gut instinct, habits, deep-seated beliefs, and emotions ( Cairney & Kwiatkowski, 2017: 2). For example, they can only pay attention to a tiny proportion of their responsibilities, so they pay disproportionately high attention to some and ignore the rest ( Baumgartner, 2017; Baumgartner & Jones, 2009). The rest is necessarily processed at a relatively low level of government, such as when delegated to civil servants, public bodies, or quasi-non-governmental organisations, often in cooperation with non-governmental organisations.

Terms such as *polycentric governance*, *multi-level governance,* and *multi-centric policymaking* describe this distribution of responsibility for action across many centres or venues for authoritative action ( Aligica & Tarko, 2012; Bache & Flinders, 2004; Cairney *et al.*, 2019; Hooghe & Marks, 2003; Ostrom, 2010). It results from a combination of *choice*, such as to use a constitution to distribute policymaking responsibilities across multiple venues, and *necessity*, when governments seek cooperation to deal with the practical limits to their power or resources ( Cairney *et al.*, 2019: 9–10). Further, the lack of coordinative capacity in one single centre, and pervasiveness of many ‘centres’, helps to explain why the rules and norms, networks, ways to understand policy problems, and windows of opportunity for policy change, differ within or across a political system ( 2019: 11–12). The pursuit of within-government collaboration reflects this need to support many venues of decision-making *and* require them to cohere.

***We need collaboration to foster policy coherence and policymaking integration***

The spread of responsibilities to address problems leads to multiple responses that – in the absence of collaboration or coordination - lack coherence or integration. There is a pressing need to coordinate responses, but no single model to do so. The absence of one centre of authority rules out a simple top-down response. The presence of multiple more-or-less autonomous organisations requires more voluntary collective approaches. Concepts to describe general aims include *collaborative governance*, *institutional collective action*, *mainstreaming*, *whole of government responses*, *joined-up government*, and *policy integration* ( Cairney *et al.*, 2019) Further, terms such as *systems leadership* reflect the limited value of hierarchical CEO leadership models, while terms such as *professionalism* and *followership* emphasis the value of different kinds of – often long-term - participation beyond the idea of instant heroic action ( Cairney & Kwiatkowski, 2017).

These accounts all share the same sense that collaboration is a ‘necessity’ rather than merely an ‘opportunity’ ( Sorrentino & Simonetta, 2012: 189). However, different approaches seek to relate this problem, and potential solutions, to different reference points including: aspirational government stories, aspirational academic stories, and policy theory-informed stories of the real world.

***Aspirational government stories of rationalism and democracy***

Aspirational stories in the European Commission often relate to evidence-based policymaking and orderly processes in which policymakers make decisions via a series of well-defined stages. The JRC uses images of a simple predictable policy cycle and its Better Regulation approach to describe and seek to get closer to such policymaking aspirations ( Listorti *et al.*, 2020: 1561). If so, the search for advice on collaborative policymaking could relate to boosting those elements, including to collaborate to improve the supply of policy relevant knowledge, and to improve necessary collaboration in relation to distinct stages of a policy cycle, including to define the problem then formulate solutions. For example, the JRC seeks to foster collaboration to improve key functions: to define policy problems then formulate technically and politically feasible solutions. The aim is to work together, across DGs, to generate meaningful understanding and agreement on what the problem is before initiating the drafting and design of potential solutions. This exercise is essential to address complex policy problems that are multi-faceted and cross traditional sectoral boundaries, promoting the need for multiple DGs to understand each other’s’ policies and perspectives - and bring in perspectives from stakeholders and citizens - before they collaborate on a new policy ( Cairney & Toomey, 2024). This search also takes place in the wider context of Commission aims to foster representative, participatory, and deliberative democracy to live up to EU ideals.

In that context, our grey literature search contains multiple reports by the European Commission on the aspiration of collaborative policymaking, primarily by the JRC but also the DG for Research and Innovation (e.g. Diels *et al.*, 2024). Examples include reports that: emphasise collaboration between multiple departments and/ or between policymakers, stakeholders, and experts (e.g. Arto *et al.*, 2018; Cachia *et al.*, 2024; see also Joint Research Centre, 2002), encourage collaboration across regions ( Rakhmatullin & Hegyi, 2021), describe the benefits of specific activities to collaboration (e.g. Midtkandal & Rakhmatullin, 2014 on peer review), or envisage ‘super collaborative government’, aided by new technology to connect public services to citizens ( Vesnic-Alujevic & Scapolo, 2019: 17). Further, for example, Cave *et al*. (2014: 61) describe Intelligent Transport Systems (ITS) as ‘an area that requires and has a long tradition of close collaboration with other DGs’, as well identifying general relevant factors including a sense of clear ‘ownership’ among one DG for each task ( 2014: 71), a common language and ‘toolkit’ for joint work ( 2014: 90).

Some JRC work connects this agenda to collaborative governance, such as to exhort policy actors to use multiple tools to boost collaboration inside and outside of government (e.g. Bianchi *et al.*, 2024). Initiatives may include: ‘participatory foresight’ tools to co-narrate visions for the future with citizens and stakeholders ( 2024: A21) ‘orchestration’ methods for (a) departments, to ‘make each department take more ownership so that they can design and implement a shared vision together’, boost collective capacity and clarify key roles, and (b) levels of government, to foster similarly high ‘interterritorial collaboration’ ( 2024: A24); and, codesigning policy solutions with researchers and citizens ( 2024: A25).

Similarly, Matti *et al.* (2022: 20) provide relatively detailed advice on the use of time, space, and facilitation methods, according to the following five principles for the ‘co-creation’ of policy by policymakers and stakeholders (including citizens):

1. *Clear scope and purpose*, to establish a common focus and manage expectations. Relevant factors for discussion include: framing the problem (its size, urgency cause, and who it affects), identifying a desired outcome, establishing who is responsible for what tasks, identifying knowledge and skills gaps, and agreeing on next steps.
2. *Establish and communicate expectations,* including to map existing policies and participants and produce a ‘hierarchy of objectives’ for the group.
3. *Foster inclusive ‘collective intelligence’,* in terms of ‘individual expertise’, representation of ‘relevant stakeholder groups’ and ‘diversity of perspective’.
4. *Tailor participatory methods to the group.* There exist many methods to foster participation in groups, which can be adapted to be used in this new context.
5. *Foster a ‘systemic perspective’.* A ‘holistic, systemic perspective’ describes the interconnectedness of elements of policy problems as well as the need to avoid ‘linear’ or ‘tunnel’ thinking.

***Aspirational academic stories of democracy and consensus-seeking***

The aspirational academic literature is more likely to focus on collaborative policymaking as a vehicle for improved democracy, such as to boost deliberation and stakeholder participation to legitimise and inform policy and policymaking:

‘The term ‘‘collaborative governance’’ promises a sweet reward. It seems to promise that if we govern collaboratively, we may avoid the high costs of adversarial policy making, expand democratic participation, and even restore rationality to public management’ ( Ansell & Gash, 2008: 561).

**Figure 2.**
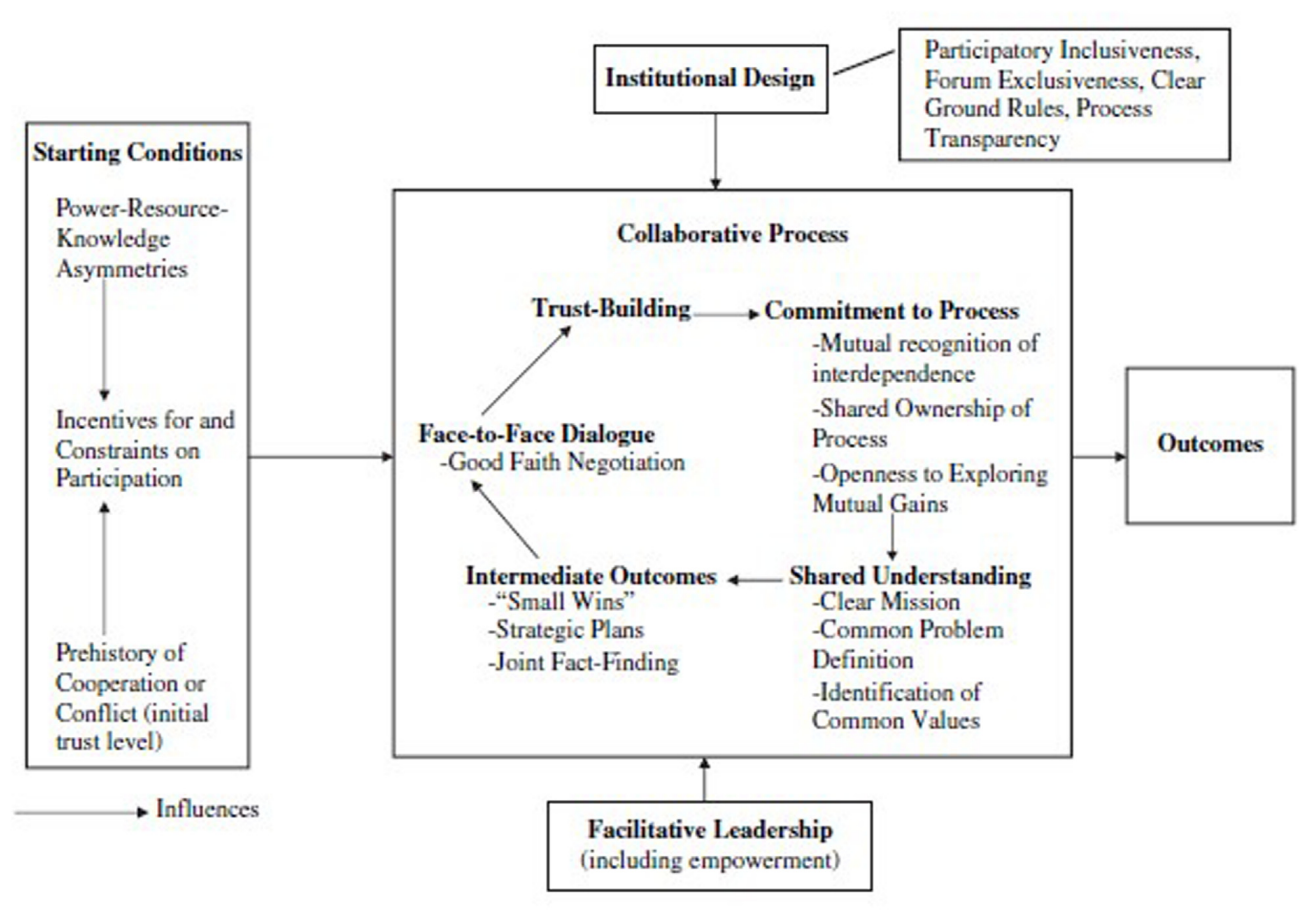
Ansell and Gash ‘Model of Collaborative Governance’. Source: Ansell and Gash (2008: 550).

Many accounts also equate improved democracy with decentralisation, in contrast to a more technocratic ideal that situates most decision-making in one centre of authority. Collaboration offers greater benefits to policymaking and outcomes than ‘managerialist’ approaches characterised by unilateral decision-making informed by narrow expertise ( Ansell & Gash, 2008: 544). This literature features advice on how to use collaboration instrumentally, to help perform or improve an essential policymaking function, as well as to help uphold key democratic principles. For example, Ansell and Gash’s (2008: 550) Figure 2, reproduced below, draws on 137 studies to describe key elements of collaboration, including:

- *Starting conditions* to attend to, such as if participants have a history of conflict, some are undermined by power imbalances or limited resources to invest, or there are other disincentives to collaborate, such as when actors favour other venues ( 2008: 555).
- *Facilitative leadership,* to bring actors together, empower them to collaborate, facilitate discussion, mediate conflict, and make sure that the process and outcomes are seen as credible and legitimate (varying roles can include the ‘professional mediator’ employed to lead, or ‘organic leader’ emerging from collaboration) ( 2008: 550–55).
- *Institutional design* or rules to foster ‘procedural legitimacy’ as well as tangible outcomes that stakeholders support. Rules should ensure an ‘open’ process that seeks to produce a wide spectrum of stakeholder inclusion, give contributors a ‘fair hearing’, seek a feasible amount of consensus, and set a deadline for key outputs ( 2008: 555–7).
- *The collaborative process itself.* Essential requirements include: (1) ‘face-to-face dialogue’, which is ‘at the heart of a process of building trust, mutual respect, shared understanding, and commitment to the process’, (2) ‘trust building’ (including preparatory work when actors have a history of conflict), (3) ‘commitment to the process’, or a sense that participants jointly ‘buy into’ and ‘own’ the process and will respect the results, (4) a ‘shared understanding’ of realistic objectives for the collaboration, and (5) beneficial ‘intermediate outcomes’ (‘quick’ or ‘small wins’) to build momentum and incentives to continue ( 2008: 561).

This general account of collaboration is a key feature of (or reference point for) the studies we include from the Web of Science search. They describe the potential benefits of collaborative policymaking if designed, supported, or executed well. These benefits can be broadly instrumental, to help get something good done well, and/ or normative, to foster fair and democratic policy processes ( Clark, 2021: 200; Kim & Siddiki, 2018: 160). Benefits may multiply if policymakers or stakeholders from each sector engage to supply multiple perspectives to a joint agenda ( Jansen *et al.*, 2015; Vandenbussche *et al.*, 2020). Or, the broad pursuit of collaboration is driven by a vague sense of policymaking complexity that should be addressed through collaboration inside and outside of government ( Hysing, 2022; Woo, 2021). Studies identify the potential to understand complex problems, identify innovative solutions, generate ownership of solutions, and strengthen key aspects of deliberative, participatory, or representative democracy ( Sørensen *et al.*, 2020: 532). They also share a focus on essential facilitators of collaboration, such as trust. We break these aspirations into the following six elements.

*First, there is a clear need or impetus to collaborate (inside and outside of government)*. Many policy initiatives involve the routine need to secure agreement across government and among public and private actors, with benefits including knowledge sharing and learning ( Arku & Oosterbaan, 2015; Cyr *et al.*, 2023: 201). Instrumental imperatives include to reduce the ‘negative effects of urban freight transport’ on cities, citizens and the environment ( Fossheim & Andersen, 2022: 1325), foster ‘give and take’ between government and business ( Woo, 2021: 221), and reduce the costs of conflict and benefits of policy knowledge and policymaking coordination ( Bramwell & Sharman, 1999: 392). Democratic imperatives include to achieve a suitable balance of power between policymakers and citizens ( Taufiq *et al.*, 2022 refer to Habermas’ ideal-type ‘communicative rationality’).

*Second, trust is essential*. Trust involves ‘a belief in the reliability of other people, organizations, or processes’ ( Cairney & Wellstead, 2021: 1), or ‘faith or confidence in another’s propensity to keep promises, to negotiate honestly, to show respect for other points of view, and to express some level of concern for the welfare of others’ ( Leach & Sabatier, 2005: 492). For example, actors need some level of trust in other actors’ commitment to collaboration. A simple calculus of trust may be based on previous behaviour, to relate the risk of investing in futile collaboration to the potential benefits of things going well. Trust may be boosted by formal and informal rules to incentivise collaboration, or if participants share the same beliefs (including their personal values and interpretations of information) or otherwise see others as their allies ( 2005: 493–4)

There is a general assumption in included studies that collaboration and trust should be mutually reinforcing, partly because common explanations for trust and collaboration overlap considerably, including the extent to which actors: (1) share similar beliefs, and (2) have seen the rewards of working together in the past ( Ingold *et al.*, 2017; Ingold & Fischer, 2014: 89). Further, suitably high levels of trust are essential to meaningful voluntary cooperation, Then, a rewarding process of cooperation may ‘help to build trust and social capital among policy actors’ which can lead them to propose win-win solutions that are ‘more legitimate and easily implementable’, and therefore reduce the ‘transactions costs’ associated with less trust (such as the costs to formalise, monitor, and enforce agreements) ( Koebele, 2021: 615). Ideally, participants engage enough to build up mutual trust, then understand and learn from each other, make choices that all accept or respect, and become more committed as the rewards from this process are increasingly clear ( Koebele, 2020: 731, citing Emerson *et al.*, 2012). Or, participants may identify extrinsic motivation, such as to see voluntary collaboration and trust building as a good alternative to government regulation ( Hysing, 2022: 207).

*Third, collaboration provides benefits to participants*. Aspirational studies state that meaningful collaboration will have benefits for the participants, such as to empower non-governmental actors to influence the policies that affect them ( Fossheim & Andersen, 2022: 1325) or at least persuade them to take responsibility for their role in policy processes and outcomes ( Vernon *et al.*, 2005: 340). Empowerment may take place via ‘authentic dialogue’ that is not undermined by power imbalances between: citizen participants ( Taufiq *et al.*, 2022: 1516), policy professionals and service users ( Jansen *et al.*, 2015: 75), large philanthropic donors and policy actors in recipient countries ( Fischer & Strandberg-Larsen, 2016: 359), or civil society and business stakeholders, to avoid consultation exercises becoming cosmetic and designed to legitimise choices already made ( Edwards & Moss, 2022: 520). New forms of engagement may offset the governmental or other hierarchies present in more formalised relationships ( Cyr *et al.*, 2023: 206). The potential benefits for citizens or stakeholder involvement can feed into wider aspirations for ‘rebuilding a new generation of more just and democratic systems’ ( Seibicke, 2024: 4). Crises may also provide a renewed impetus for collaboration, problem solving, and innovation ( Bourdin *et al.*, 2024). Alternatively, public managers may use collaboration more strategically, to boost their capacity and support for policy outputs initiated from the ‘top down’ ( Hysing, 2022: 203).

*Fourth, we need to connect the benefits of collaboration to the incentives to collaborate*. Studies connect potential benefits of collaboration to incentives or payoffs. They include a relatively abstract sense of empowerment to contribute to common aims through meaningful deliberation ( Fossheim & Andersen, 2022: 1325; Taufiq *et al.*, 2022: 1517; although intrinsic and extrinsic motivation for stakeholders remains under-researched, Seibicke, 2024: 5). Actors may engage in collaboration if they think they ‘can help make a positive difference’, collective aims align with their individual or group values, and their engagement is in ‘their community’s interest’ ( Webler *et al.*, 2003: 105). This perception of empowerment relates strongly to the perception that ‘the collective deliberation process treats parties fairly and consistently’, which – especially in high conflict situations, or where there are significant differences of belief or opinion - may be facilitated by a ‘respected mediator’ ( Siddiki & Goel, 2017: 257). Benefits may also relate to learning: ‘extended, face-to face deliberation’ may help participants to ‘learn about the problems at hand and better understand one another’s unique needs and values’ and perhaps even encourage some convergence in beliefs ( Koebele, 2021: 615).

*Fifth, collaboration provides benefits to policymaking*. Studies describe the high potential for meaningful collaboration to produce improvements in policymaking. It may boost public sector capacity to engage with policy problems and the reputation of organisations (if viewed as trusted candidates for cooperation) ( Seibicke, 2024: 6; 11). An increase in policymaking capacity may boost ‘the innovation of joint solutions’ ( Fossheim & Andersen, 2022: 1325) or ‘more innovative public sector’ ( Arrona *et al.*, 2020: 119). Terms like ‘learning, synergy and commitment’ describe what better policymaking would involve: the ability of actors to learn from each other to update their beliefs and approaches, find common ground and aims, and invest or gain ownership from the process, to boost stability and delivery ( Arrona *et al.*, 2020: 129, citing Ansell & Torfing, 2014):

‘Empirical evidence shows that multiactor engagement can lead to new ways of seeing and framing problems, of thinking and agreeing on solutions, of coordinating actions and of creating a sense of ownership that facilitates the implementation of innovation and generates public value … Learning occurs as the product of intense interactions among people with different knowledge and visions, leading to new ideas or to reframing old ones. Synergy refers to the process of joining capacities and resources to carry out innovative actions. Lastly, collaboration can generate commitment and a sense of ownership among actors that enables consensus-building and support for the implementation of innovative solutions’ ( Arrona *et al.*, 2020: 123).

This support may be intrinsically valuable, or instrumentally valuable when inducing non-governmental actors to help to produce policy outcomes ( Hysing, 2022: 205). For example, governments may see partnership working as a more effective alternative to the top-down management of the public sector or direct regulation of policy targets ( Erikson & Larsson, 2022: 824). However, few go as far as Zhang *et al*. (2024: 9) to seek to identify an ‘optimal combination of stakeholder engagement’ for each part of the process.

*Sixth, collaborative policymaking brings benefits to policy.* The general assumption, or theory of change, is that well designed and executed collaboration will improve policymaking and therefore the quality of policy ( Siddiki & Goel, 2017: 254). Improved quality can relate to the evidence-informed understanding of a problem and creativity to address it, a policy tailored to local context, and the policy’s appeal to stakeholders or citizens which boosts ownership or at least a sense that it represents a well-discussed plan ( Taufiq *et al.*, 2022). Collaboration may also foster smoother implementation processes by better connecting the actors making and implementing policy ( Ansell *et al.*, 2017: 467–9). An exemplar of an optimistic account of collaboration is that:

‘Collaboration facilitates a joint exploration of policy problems that allows the relevant and affected policy actors to agree on novel ways of defining the problem that both emphasise its urgency and make it solvable. It spurs a constructive use of scientific knowledge in processes of mutual learning and creative problem-solving. Hence, when the participating actors have developed sufficient trust in one another, they will stop using scientific results as political weapons and begin to craft and circulate new ideas and disruptive solutions that are further improved through critical scrutiny, cross-fertilisation and integration … Collaboration also enables a careful evaluation of alternative solutions through a joint assessment of potential risks and gains that may draw on an experimental testing of prototypes … Last but not least, the opportunity for relevant and affected actors to participate in the design of innovative solutions will create a sense of joint commitment to and responsibility for the implementation of the innovative policy design. A joint ownership over innovative policy solutions will help to prevent ignorance, passive resistance and direct sabotage on the part of street-level bureaucrats, and encourage target groups and private stakeholders to explore the possibilities for transforming the logics of the societal subsystems in order to support the realisation of shared policy objectives’ ( Ansell *et al.*, 2017: 476).

Wider benefits to society relate to an ability to ‘restore trust in government, foster public engagement, and further the legitimacy and effectiveness of public actions’ ( Hysing, 2022: 201). Indeed, the European Commission may use collaboration – across government, with experts, and with stakeholders - to project ‘procedural legitimacy’ ( Hensell, 2022: 153–5).

***These aspirations can also be found in the grey literature***

Our general impression from the grey literature search is that report authors are just as likely as academic journal authors to recommend collaborative policymaking without describing what it is or how governments should do it ( Eurofound, 2006; Parmitha, 2016). Many state the general potential benefits, such as to:

- Boost policy learning or trust between countries ( Atlantic Council, 2019).
- Build a ‘common understanding and strategic vision’ to aid ‘long-term collective reflection and discussion’ between countries and international organisations, such as to help anticipate and respond to financial, public health, or climate crises ( Cheng *et al.*, 2018: 15–16; 24–25; European Centre for Disease Prevention and Control, 2018: 12; Saidi, 2023; Stauffer *et al.*, 2023).
- Work with the private sector and NGOs to tailor policies to local context ( RSIS Centre for Non-Traditional Security Studies, 2014: 3). Terms such as ‘cross-sector development partnerships’ (CSDPs) and ‘Cities Climate Finance Leadership Alliance’ describe collaborations between public, private, and third sector organisations. Their value relates to the credibility and visibility of collaborative projects, sharing resources, improved networks, trust and learning between partners, and ‘the synergistic value’ of projects that would not occur from independent action ( Climate Policy Initiative, 2023: 10; Kindornay *et al.*, 2014: 2).
- Foster ‘agile policymaking’ characterised by collaborative innovation to produce policy solutions and set common standards across countries ( Davis *et al.*, 2022: 18).
- Share research knowledge across: government ( EMCDDA, 2012: 4), the public and private sectors ( Dondo *et al.*, 2022; Hanin *et al.*, 2023; Public Policy Forum, 2013), or between researchers and policymakers ( Hudson & Marquette, 2015; Productivity Commission, 2010: 228), including to provide ‘decision support’ when using technical models ( Manheim *et al.*, 2016: 4). For example, widespread collaboration to share research is essential to ‘advocacy and knowledge transfer’ given that mainstreaming agendas such as the Green Deal involve a ‘multitude of actors at all levels of government in a system of multilevel governance’ ( Klika, 2021: 14; see also Scott, 2023; Diels *et al.*, 2024).
- Use collaboration to address ‘complex governance’, since ‘unnecessarily fragmented and disconnected systems lead to inefficiencies, redundancies, and gaps in addressing needs and providing services’ ( Resilient Puerto Rico Advisory Commission, 2019: 39).

Multiple reports also identify the value of ‘co-creating’ policy with citizens and stakeholders as a ‘governance tool’ which: ‘fundamentally affects the success or failure of any public intervention. It is a way to understand the needs and interests of the different players involved in a complex problem, gather their knowledge and insights and act accordingly for the successful implementation of the policy or service in question’ ( Dixon *et al.*, 2021: 2).

### Empirical stories: complex policymaking systems do not seem to be conducive to such collaboration

We use Cairney’s synthesis of policy theory insights to show how they contribute to a messier story of how policymaking really works ( Cairney, 2020; Cairney, 2024). This synthesis provides an overarching theoretical and empirical story of bounded rationality ( Simon, 1976) and policymaking complexity. Collaboration takes place in a policymaking environment over which policy actors have limited understanding and control. It suggests that what we require – policy coherence and policymaking integration - does not match practical or political reality. Theories demonstrate the impact of messier reality when:

- Policymaker attention lurches from issue to issue, often before addressing the problem in a meaningful way (or harvesting the fruits of collaboration).
- Policymakers necessarily must act – often quickly and decisively- despite uncertainty about the problem and likely effect of action (which may limit their reliance on lengthy collaborative processes).
- Policy actors compete to define and address problems, and cooperation is never guaranteed. They pursue their beliefs and protect their positions, often at the expense of collaboration.
- The distribution of policymaking responsibilities in political systems often reflects demands for political or territorial autonomy rather than a technocratic search for optimal policymaking. If so, the push for collaboration may be perceived as interference or part of turf wars.
- The ‘usual ways of doing things’ undermines new approaches. Rules, norms, and practices in one venue may conflict with another (making it difficult to set new ground rules for collaboration).
- There are good reasons to specialise to deal with technical issues, or close off policy communities to foster small-scale cooperation. Such defendable arrangements produce silos that make sense in their own context but undermine cross-community action. What makes sense in relation to one policy community makes little sense in another.
- Technically political solutions can be politically infeasible, and vice versa. If so, collaborative work may help to create responses that seem feasible to participants but are difficult to sell to external audiences (this summary is reproduced from Cairney’s synthesis of insights - from sources including Baumgartner & Jones, 2009; Kingdon, 1984; Weible & Heikkila, 2017; Cairney, 2020; Cairney, 2021; Cairney *et al.*, 2022; Jordan & Halpin, 2006 – in Cairney, 2024; Cairney & Toomey, 2024).

Indeed, audiences such as the JRC provide stories and images to describe this messier policymaking reality, and to try to understand the barriers to more effective policymaking ( Cairney, 2022; Topp *et al.*, 2018), at the same time as producing more aspirational models. In that context, the search for lessons may involve the pursuit of ways to improve existing processes rather than expect fundamental transformations towards collaborative policymaking ideals. To that end, the following section identifies routine barriers to collaboration to aid the realistic assessment of new advice.

### Combining aspirational and real-world stories: what are the routine barriers to collaboration?

Most of our included empirical studies recognise this gap between the intended benefits of collaboration and the factors that may hinder progress, as follows.

First, policy problems may not be amenable to successful collaboration. The policy problems that require the most collaboration are often associated with the need for most ‘transformation’, such as from a high to low carbon energy systems as part of climate change policy. Yet, such ‘transformations defy planning’ and ‘involve complex systems’ that are difficult to understand far less change ( Jacob *et al.*, 2021: 4). As such, the high *need* for integration and change does not signal the high *possibility* of effective collaboration.

Second, broad conceptual aspirations may not translate into concrete outcomes. Trust may be essential but in short supply ( Cyr *et al.*, 2023: 203), especially if there is a lack of collaboration on which to build, negative experiences of collaboration in the past, or value-based polarisation that may be worsened by ill-designed interaction ( Ansell *et al.*, 2017: 476). Further, ‘learning’ is a vague term to describe a range of activity, not all of it good, that we don’t fully understand ( Koebele, 2019: 242).

Third, collaboration takes an investment of time and resources, to engage with problems for the long term and seek ‘deep learning’ that involves gathering new knowledge and fostering eventual shifts in norms and beliefs ( Siddiki & Goel, 2017: 257). If so, and given there is no guarantee of reward, participants may see collaborations as ‘too time- and resource-intensive to sustain’, especially when the benefits are relatively abstract (e.g. to maintain democratic or policy process norms), the forum is perceived to be a talking shop, or rewards involve ‘least-common-denominator’ solutions rather than the innovative breakthroughs advertised in optimistic accounts ( Ansell *et al.*, 2017: 476; Hysing, 2022: 207; Koebele, 2021: 615).

Fourth, many barriers to meaningful collaboration may be group specific:

- Elected policymakers may be reluctant to go beyond symbolic policymaking, share power, compromise their beliefs, upset their favoured interest groups by widening the net of collaboration, or slow down policymaking to collaborate properly ( Ansell *et al.*, 2017: 479–81; Ingold & Fischer, 2014: 96).
- Public bureaucracies may treat wider collaboration as an expensive distraction from core business ( Sørensen *et al.*, 2020: 533). They may express ‘limited commitment to long-term relationships’ without a meaningful incentive to engage ( Sorrentino & Simonetta, 2012: 198). Risks to public organisations, when collaborating with non-governmental actors, include ‘agency capture, mission drift, loss of organisational autonomy, costs, difficulty evaluating results, and problems with upholding public accountability’ ( Hysing, 2022: 204). For example, it may become difficult to identify who is responsible for what and why, which is a problem when elected and unelected policymakers have distinctive roles in relation to public accountability ( Erikson & Larsson, 2022: 831).
- Powerful non-governmental actors may prefer to influence policy informally or in relatively secret meetings than show their hand in formal collaborative spaces ( Taufiq *et al.*, 2022). Some may be reluctant to share knowledge with others if they are competitors in other contexts ( Arku & Oosterbaan, 2015: 372). Or, their involvement may be conditional on the participation of other groups ( Zhang *et al.*, 2024: 8). The ‘apathy’ among key actors may be difficult to counter if a key driver of collaboration is voluntary rather than enforced action ( Vernon *et al.*, 2005: 341).
- Less powerful stakeholders recognise that they contribute to a process out of their control, such as when consulted after an agenda is set by elected policymakers ( Edwards & Moss, 2022: 522).

From our grey literature search, Dixon *et al*. (2021: 2) make a similar assessment of the ‘infancy’ of ‘co-creation’ between governmental and non-governmental actors:

‘It is too often one-off and fragmented. It is pursued as an end in itself rather than a means for better serving users. Its definition is stretched to encompass almost any activity, including a simple brainstorming meeting with stakeholders as long as white boards and “post-it” notes are used. Its results are elusive because of its organic and serendipitous nature and cannot be evaluated through standard business practices such as measuring return on investment in particular when dealing with societal challenges. For sure, there are standardised methodologies and techniques, job profiles and technological tools for assessing progress, but they are not widely adopted’.

Such barriers, combined with the potential for ‘collaborative policymaking’ initiatives to seem tokenistic, suggest that we should not *assume* that the potential benefits of collaboration are widely perceived, shared, or realised. On the one hand, studies often find examples of substantive benefits from the perceptions of interviewed participants ( Koebele, 2020; Koebele, 2021; Vernon *et al.*, 2005: 341). On the other, participants find it difficult to know how fruitful collaborations were. For example, Vandenbussche *et al.* ( 2020: 9) find in a follow-up interview that a participant had previously expressed ‘wishful thinking’ about progress.

Overall, these cautionary tales convey a gap between positive aspiration and practical reality, and this gap helps to make sense of advice on collaborative policymaking. The advice may be the same each time, but will mean something different to audiences seeking order or expecting mess. Policymakers may seek aspirational advice to promote an ideal, pragmatic advice to address reality, or some combination of both.

## What would good collaborative policymaking look like? General features of policymaking design

Some academic studies describe the general or contextual importance of the design of institutions (in other words, rules) to support collaboration. The aim may be straightforward, to ask a three-part question: what is the problem, what should we do, and how should we do it ( Vernon *et al.*, 2005: 328)? However, the design of institutions to support that process is more complicated, since it situates those questions in a wider context to establish who is ultimately in charge and how new participants fit in. Here, institutions are both the formal, written, and well understood rules of engagement as well as the informal and unwritten rules, norms, or practices that may not be communicated in writing or verbally ( Ostrom, 2007; Woo, 2021). Only some of these rules are in the gift of the designers of new collaborative arrangements.

Many formal institutional design features can be *largely outside of the control of collaborative policymaking advocates.* First, they can include the overarching nature of political systems, such as if majoritarian political systems are less conducive than consensus democracies to collaboration, when authoritarian systems have a limited tradition of sharing power compared to liberal democracy settings, or if engaging in ‘clientelistic’ systems based on dispensing favours to certain groups ( Ansell *et al.*, 2017: 481). Second, policy networks or subsystem dynamics can be categorised as ‘adversarial’ or ‘collaborative’, with the latter associated with more common ground on beliefs and a greater propensity to seek cooperation, which is broadly conducive to learning from scientific evidence rather than using debate on evidence to fuel tense exchanges ( 2017: 476, drawing on Weible, 2008; Weible & Sabatier, 2009; Weible *et al.*, 2010). Third, political system rules may relate to managing ‘multi-level governance’ across the EU, such as when the EU formally obliges member state or subnational government action (e.g. to support participatory or collaborative governance during policy implementation) but also signals informal flexibility regarding when and how to carry it out ( Koontz & Newig, 2014: 594–5).

Other design features can be *relatively abstract aims or principles* to encourage rather than ensure collaboration. Aims can include *inclusiveness* to foster democratic principles and pragmatic ways to gather as many insights as possible, and *interdependence* when people may need or want to work together. However, these factors contribute to unclear causation. For example. a large and inclusive network may signal that most participants are welcome to contribute, but a small and exclusive network allow fewer people to use higher interactions and experience of collaboration to build trust and find a rhythm of working together. Similarly, interdependence may signal the need rather than desire to work together, which might be conducive to good working relations if people accept the inevitability of the relationship, or not if they resent their reliance on others. Often the assumption is that meaningful collaboration comes from fostering democratic principles and intrinsic motivation rather than exclusivity and forced relationships. Some also suggest that inclusiveness boosts the influence of participants compared to more exclusive arrangements ( Fossheim & Andersen, 2022: 1325). However, stakeholders may also be more engaged if that process ‘is viewed as being the only venue in which they can address their concerns’ ( Siddiki & Goel, 2017: 257). Further, some studies identify the surprising gap between aspiration and reality, such as when ‘collaborative approaches’ might help to ‘mitigate conflict’ (or the ‘convergence in some beliefs’ between otherwise competing coalitions) but without necessarily boosting reliance on evidence-informed approaches to policy discussion ( Weible & Sabatier, 2009: 195).

More detailed studies focus on the specific ways to *institutionalize collaboration* that are more likely to be in the gift of organisers ( Koebele, 2021: 619). Although they do not have a how-to guide in mind, they offer a sense of the factors to attend to (which we interpret below), such as the ability to establish new informal working groups with the freedom to foster meaningful collaboration ( 2021: 619–20), or the use of external actors to provide an impetus for new discussions ( Ansell *et al.*, 2017: 481–2). Such design rules may serve to address imbalances of participant power in different ways, such as to use ‘different definitions of consensus, ranging from a minimum standard of acceptability to unanimous support from all participants’ ( Koebele, 2020: 730). Further, designers may attend to the ‘life cycle stages’ of collaborative arrangements: (1) ‘activation’ (defining the problem and relevance of participants), (2) ‘collectivity’ (gathering resources, setting norms and rules, fostering ‘working as a whole’ and sharing credit), (3) ‘institutionalization’ (reaching a point where the group’s constituent parts cohere, to act collectively in relation to external actors), and (4) ‘stability, decline, re-orientation, and re-creation’ (when the activities of the network fluctuate in relation to participant attention or engagement) ( Siddiki & Ambrose, 2023: 623, citing Imperial, 2023).

Some studies provide a useful checklist to identify how a collaborative project lives up to essential requirements, including:

1. *Scope*, which describes the extent to which key stakeholders are represented, stakeholders perceive a benefit to engagement, and the facilitator is invested in the process and outcome, as well as the numbers of actors involved in each activity, and initial levels of stakeholder agreement.
2. *Intensity,* including a perception among stakeholders that collaboration will shift their views and actions, how and how often they are involved, the effectiveness of information sharing, if participation involves information sharing or direct discussion, if the dialogue is honest and respectful and conducive to trust-building, if exchanges help stakeholders to gain more understanding of the issue or other stakeholder views, and if the facilitator fosters collaboration rather than top-down discussion.
3. *Degree to which consensus emerges,* including the development of realistic expectations on agreement and on the feasibility of solutions, common views on aims and solutions, reaching meaningful common ownership of solutions when there are power imbalances between participants, and a commitment to implement the results ( Bramwell & Sharman, 1999: 395–401).

Studies may focus on *perceptions of good collaborative design*: ‘1. the group process is perceived as being fair and consistent for all involved parties; 2. all critical stakeholders are engaged in the process; and 3. the group has at least one participant who mediates conflict and is held in high esteem’ ( Siddiki & Goel, 2017: 258). Studies may also focus on the design of collaborative approaches to connect actors involved in policy design to implementation *.* Collaborative design fosters ways to gather knowledge on local variations in policy context and the factors likely to influence policy delivery, gauge the skills and resources required for delivery, provide an ‘early warning system’ about likely problems, and prompt new ideas by stakeholders and citizens (if they are treated with respect) ( Ansell *et al.*, 2017: 478).

## Discussion: Actionable lessons from academic and practitioner research

A subset of articles and reports describe their findings in ways conducive to actionable advice. They include not only grey literature reports with direct advice (e.g. Dixon *et al.*, 2021), but also interpretive scholarship that could inform advice. The latter suggests that everyday ‘mundane’ practices can reflect profound issues such as the power dynamics between actors, represent a way to manage day-to-day activity with “‘small’ steps” while facing “’big’ problems”, and highlight elements conducive or unhelpful to meaningful collaboration ( La Grouw *et al.*, 2024: 6; 15–17). However, since vanishingly few academic studies provide explicit recommendations, our advice translates findings to recommendations in ways that the original authors would not have anticipated. In other words, we synthesise insights from these studies to identify key features of collaborative policymaking, and situate the outcome in ‘Discussion’ to signal our role in producing these results. We distil potentially actionable advice into five categories: collective planning, identity, practices, spaces, and skills ( Table 2).

**Table 2.** Five features of collaborative policymaking.

**Collective planning:** **Prepare to collaborate**	Design rules for inclusion
	Signal respect for participants
	Surface values and beliefs
	Identify how to support learning
	Clarify and explain routines
	Identify what can go wrong
**Collective identity:** **Create a clear purpose**	Set a boundary around your work
	Foster a common vision and language
	Define success for the collaboration
**Collective practices:** **Use creative methods**	Visualise and map the problem and network
	Use design techniques for policy and reflection
**Collective spaces:** **Create new forums**	Use working groups to support core groups
	Use informal venues for experimentation
	Use policy labs to co-design solutions
**Collective resources:** **Clarify roles and skills**	Ensure sufficient process expertise
	Use external facilitators
	Secure policy sponsors or champions
	Maintain wider political support

### Collective Planning: Do your research to prepare for in-person meetings

Effective collaboration involves face-to-face discussion to boost trust, learning, and cooperation. However, the following essential activities, to design and communicate the value of collaboration, take place before meetings.

***Design and justify your rules for inclusion, addressing trade-offs regarding group size and diversity***

First, treat collaboration as a core guiding principle. Collaboration may combine with principles such as ‘social wellbeing’, ‘equity and inclusiveness’, and ‘transparency’ to support regeneration ( Resilient Puerto Rico Advisory Commission, 2019: 10). Or, it may combine with principles of ‘capacity’, ‘leadership’ and ‘accountability’ to foster ‘long-term thinking across the EU’ ( Dirth, 2024: 1).

Second, communicate a strategic approach to, and expectations for, collaboration *.* Explain why collaboration would be preferable to unilateral action. Establish its purpose, foster necessary cross-government support, induce voluntary action among all key participants, and clarify key elements with participants ( Hysing, 2022: 218). Key aims may be to deliberate long enough to produce a shared language or understanding, build enough trust to produce common aims, and secure enough capacity to support this work, including fair procedures, effective leadership, and a repository for shared knowledge ( Kim & Siddiki, 2018: 161, citing Emerson & Nabatchi, 2015). If so, signalling that a collaboration is designed to last for (say) five years could ‘bolster trust’, since it appears to grow ‘among participants who plan to interact with the other members of the partnership’ for a substantive amount of time ( Leach & Sabatier, 2005: 499). That said, some groups may prefer the sense that they can get something useful from collaboration without having to over-commit in advance ( Koebele, 2020: 742).

Third, establish a desired Goldilocks level of participant activity with respect to diversity. Diversity can relate to *representation*, such as of a government, government department, or stakeholder, and *knowledge* or *beliefs* about the policy problem ( Kim & Siddiki, 2018: 159). Diversity may be a normative aim relating to inclusiveness and fairness, and instrumental aim relating to the benefits of considering many perspectives and keeping people on board. However, it comes with the risk of unintended consequences, such as if more participation produces a tipping point away from ‘consensus’ towards ‘intractable conflict’ ( 2018: 160–1). A ‘moderate level’ of participant diversity may be key to a group’s perception of procedural fairness, although ‘moderate’ is difficult to operationalise. Belief diversity is more difficult to assess without further research, since different participants may bring but not describe very different assumptions or beliefs about, for example, the primacy of scientific evidence over other sources ( 2018: 171; Hysing, 2022: 207). There is also a trade-off between immediate instrumental aims, such as to limit group size and belief heterogeneity to foster collaboration, and longer term aims to maintain external legitimacy, such as to make sure people feel included and treated fairly in networks ( Leach & Sabatier, 2005: 499).

Fourth, ensure that collaboration design is appropriate to the task, establishing clear management, rules, and incentives to participate. Define the collaboration’s objectives to inform the appropriate roles and tasks of participants ( Kindornay *et al.*, 2014: 4). Identify core functions, such as to: establish a clear trajectory for policy collaboration (‘visioning’), enable and support engagement among key partners (‘facilitating’, ‘supporting’), promote the results of collaboration to others (‘amplifying’), and protect wider organisational aims and values during new arrangements that can blur boundaries between responsibilities (‘guarding’) ( Eneqvist & Karvonen, 2021: 185–6).

***Signal respect for participants in the formal procedures for inclusion and collaboration. Anticipate the unintended consequences of innocuous informal rules and norms***

Simple and visibly respectful practices, to signal cooperation, can include a decision to rotate where people meet in person, or to attend to any obstacles involved in visiting a host (e.g. getting through security as a colleague or guest) ( La Grouw *et al.*, 2024). However, it is also important to make the invisible visible, to surface issues such as power imbalances between participants and the likelihood that some actors will exercise their power behind closed doors and away from formal collaboration ( Taufiq *et al.*, 2022). Further, innocuous looking factors can have disproportionate effects, such as the location and layout of the meeting space and what it signals to participants ( La Grouw *et al.*, 2024: 12), or the routinely positive terms that actors use to describe themselves and therefore how they ‘other’ their potential collaborators (e.g. professional/unprofessional and expert/non-expert, Jansen *et al.*, 2015; Vandenbussche *et al.*, 2020).

***Surface the values and beliefs of participants, to help set realistic expectations***

Establish the extent to which collaboration is possible, such as by relating policymaker or participant beliefs to the task. An excessively technocratic approach would downplay the values of participants. However, with some issues, we need to identify belief conflict between actors to anticipate the need for conflict resolution before meaningful collaboration can take place (e.g. Pettrachin, 2024 on migration policy).

A preliminary focus on problem definition should include a frank discussion of current levels of belief conflict and scope for collaboration among the actors essential to the project, which may necessitate up-front key choices rather than putting them off. For example, take the time to surface beliefs about the size, urgency, and cause of the policy problem, the role of the state in solving it, which governments should be responsible, how they should operate (e.g. to centralise or decentralise), the envisaged timescale for transformations to policy and policymaking (e.g. when seeking a ‘just transition’), and the intended recipients of benefits and costs ( Clark, 2021: 209).

These preliminary exercises may also highlight assumptions that people hold about their preferred policy processes, including fundamental issues of good governance, as well as more innocuous uncertainty about who should take charge of tasks such as policy drafting. In relation to the former, some may bring a ‘managerial perspective’ that favours ‘specialization and expertise, authority, formalization, and political neutrality’ while others bring the ‘political public values framework, which is directly linked to democracy and concepts such as participation, representation, political responsiveness, liberty, and equality’ ( Clark, 2021: 209). Advocates of public values describe wider legitimacy benefits: the perception, among marginalised groups or citizens, that they are being heard in some key venues increases support for collaborative policymaking in others ( Mewhirter *et al.*, 2024: 300).

These preliminary discussions could be supported by preparatory research to inform deliberation, including interviews with (or surveys of) potential contributors to surface their willingness and motivation to engage. Examples of key information and action include:

- If you find perceptions that collaboration will be more symbolic than substantive, establish what it would mean for each participant to feel involved and well-informed, their expectations for reasonable compromise, and what levels of transparency and decision-justification seem appropriate ( Edwards & Moss, 2022: 523–7).
- Levels of cooperation may vary according to task or function, such as if ‘power games and belief conflicts’ are most intense during the definition of the problem and selection of high or low state solutions, compared to the routine delivery of negotiated policies ( Ingold & Fischer, 2014: 90).
- If concerned about the low compatibility of beliefs between key participants, establish the extent to which they may seek to exit collaboration or see this venue as the only realistic means to negotiate on their aims ( Koebele, 2020: 730).
- Concerns about exit can be addressed by signalling a high commitment to respecting decisions made during collaboration (to ward off an incentive to seek redress elsewhere – Leach & Sabatier, 2005: 499). Or they may prompt you rely on brokers or ‘boundary spanners’ (or ‘boundary organisations’) able to operate across competing groups ( Koebele, 2020: 730; Larson *et al.*, 2009: 1020).

Such concerns about belief conflict can be addressed in relation to their perceived severity, such as if ‘the relative influence of various actors is ambiguous’, ‘stakeholders disagree over fundamental values and norms’, ‘the issues are scientifically complex, such that policy actors also disagree on the relative severity and causes of different problems’, and ‘monitoring and enforcement mechanisms are difficult or impossible to establish (such as negotiations among autonomous and highly heterogeneous stakeholders)’ ( Leach & Sabatier, 2005: 500):

‘If distrust stems from disagreement over which problems are most serious, then deliberations should begin with a period of “joint fact finding” and consensus building on the basic dimensions of the various problems. Partnerships can also pursue empathy-building exercises, such as field trips to the local businesses or environmental sites affected by the partnership’s actions. Over the long term, partnerships can commission scientific investigations to settle disputed facts’ ( Leach & Sabatier, 2005: 499).

In some cases, research may highlight the ‘devil shift’, when some actors overestimate the power and exaggerate the malicious motives of their competitors ( 2005: 499). If so, exercises could examine how to respond if these perceptions are: (1) inaccurate, prompting ‘design exercises to lead stakeholders toward accurate assessments of their relative power within the partnership by exploring each party’s best available alternative to negotiation’, or (2) accurate, prompting ‘particular attention to bolstering trust among politically weaker parties’ ( 2005: 499).

***Identify how to support the elements essential to mutual learning***

Positive learning may involve acquiring new information, translating it to a new context, sharing it across the group, then using it to contribute to cognitive then behavioural change ( Heikkila & Gerlak, 2013, cited by Koebele, 2019). Key contextual factors include initial levels of trust or conflict among participants and the impact of external political pressures ( Heikkila & Gerlak, 2013: 496–500). Factors more in the control of designers include: the technology to generate and share information; and, the rules of information sharing, such as to foster open dialogue among many actors to favour diversity of thought or tightly coordinated discussion to favour efficient discussion and low costs of coordination ( 2013: 496–500).

***Clarify and explain routines, including the mix of in-person and online collaboration***

In-person collaboration can be essential to build a tangible sense of collective endeavour and foster mutual learning ( Koebele, 2019: 253), then encourage participants to work intensively to build initial trust and quick wins. Subsequently, online meetings may become more efficient means to meet regularly and discuss and monitor progress. However, balance needs to be monitored, since problems arising during online discussions can reflect more fundamental issues, which should prompt ad hoc in-person meetings ( Cyr *et al.*, 2023: 205). Face to face meetings help to create ‘moments of negotiation on process and outcomes’ and add ‘much-needed humanity to our largely virtual relationship’ ( 2023: 205).

***Identify the practices that can undermine effective collaboration, including poor planning and vaguely described activities***

For example, Dixon *et al.* (2021: 10) identify what to avoid during processes of ‘co-creation’, including:

- unprepared meetings and ineffective ways to initiate discussion (such as a poor opening question to participants, or poor process to manage open discussion)
- blaming participants for poor meetings or outcomes
- treating exercises as one-offs
- creating unrealistic expectations
- underestimating the costs of cooperation to the participants you need to get involved.

### Collective identity: Create a clear sense of purpose for the collaboration

Effective collaboration is built on a collective sense of purpose, aided by the following actions.

***Set a boundary around your work, establishing which issues and actions are relevant***

Boundaries help to turn an overwhelmingly complex problem into a simpler task with limited aims and participants ( Sørensen *et al.*, 2020: 534). The task is to cross traditional sectoral boundaries and connect new aims in a coherent way (e.g. La Grouw *et al.*, 2024 describe the value of a geographical boundary). Establish what frames or tasks are to be included in collaborative work and what can be deemed as out of scope.

***Foster a common vision (guiding principle, strategy, objectives) and language***

Policy actors with limited formal resources, or a reluctance to use them to oblige collective action, can make ‘an emotional connection with people to exert influence through a collaborative endeavour, rather than employing bureaucratic authority’ ( Ayres, 2019 on ‘soft metagovernance’, cited by La Grouw *et al.*, 2024). For example, the use of narrative can aid the development of a ‘common vision’ to guide the collaboration, which consists of a collection of formal and informal interactions ( Ayres, 2019: 286). The trade-off is between an ambiguous vision to foster initial agreement and a clearer statement of intent to reduce the potential for the unhelpful reinterpretation of aims.

Collaboration may also benefit from an agreed language to describe common work, or at least a means to translate sectoral or professional jargon. Attend to obstacles to communication when actors use different terms and reference points, including a jargon with limited reach or the potential to use the same terms to refer to different experiences or perspectives. A new language can signal cooperation ( Gunashekar *et al.*, 2024: 27), albeit without resolving everyday tensions between professional niche languages ( La Grouw *et al.*, 2024) or a language used by practitioners but not service users ( Jansen *et al.*, 2015: 75). 

***Define success for the collaboration as well as the policy. Provide measures of progress for the group as well as longer-term policy outputs and outcomes***

While the ultimate aim of collaboration may be measurable policy outcomes, the first aim is to establish intermediate milestones for the collaborative process. Key measures can be of collaboration activity or impact in the context of a tendency for collaborative arrangements go through ‘life cycles’ of engagement ( Imperial, 2023). Examples include:

- Siddiki and Ambrose (2023) devise indicators to measure the frequency and substance of engagement, which may be useful to compare with essential periods of engagement.
- Leach *et al.* (2002: 654–6) devise survey measures of participation, including: ‘perceived effect on human and social capital’ (via information sharing and trust development), ‘level of agreement reached’ (e.g. on principles, objectives, or concrete actions), as well as perceptions or monitoring of follow-through on commitments and the collaboration’s impact on a wider network of colleagues.

### Collective practices: Use creative methods to design a collaborative vision

Effective collaboration requires participants to articulate a shared vision, which can be facilitated by the following methods.

***Visualisation methods to map your policy problem, network, and intended response***

Visualisation can prompt discussion about how actors frame problems and understand their role in policy design. Producing images can help to model what is known about a problem, identify multiple means to address it (and see how they connect), and surface trade-offs or dilemmas on the policy agenda and its delivery ( Carton & Thissen, 2009: 1994, discussing visualisation of geographical and policy maps; compare with Ahrweiler *et al.*, 2019 on ‘co-designing social simulation models’). Or, it can be part of foresight activities including the creation of scenarios to guide discussion of feasible collective responses ( Aro *et al.*, 2023).

***Design techniques to inform policy and reflect on collaboration***

Processes of policy design can be a tool for reflection as well as planning, such as to surface the difference between official images of ‘rational decision-making’ and unofficial understandings of the use of ‘intuition’, experience, informal rules, and creativity to make imaginative, ‘human-centred and future-oriented’ decisions ( Spaa *et al.*, 2022: 379–81). Reflection may also aid discussion of the improvement of processes such as scenario planning, including to ask if the process is:

- conducive to honest discussion and investment by participants
- transparent to all and not driven solely by implicit norms of one group (e.g. to only include politically feasible responses in scenario discussion)
- backed by sufficient resources (time, expertise, coordination efforts)
- well understood by participants, especially when aspects are technical
- well communicated externally
- sufficiently flexible and dynamic to keep up with changing circumstances ( Aro *et al.*, 2023: 205).

Further, such exercises may rely on high levels of trust in the scientific evidence or models used to inform scenarios, necessitating a clear ‘reporting standards’ process to inform ‘collaborative modelling’ ( Teerawattananon *et al.*, 2022: 5–6).

### Collective spaces: Create new forums to support collaboration

Formal and well-established policymaking rules and venues may be indispensable, but they can still be supported by new and more flexible spaces for collaboration.

***Working groups to support core groups, creating ‘soft spaces’ of governance to foster informal discussion and experimentation***

Using new or lower stakes forums may help governments navigate wider tensions. In some cases, high level formal cooperation is hindered by poor relations between countries (exacerbated by imbalances in their size and power), prompting organisations to use more informal working groups to bypass a sole reliance on tense formality ( Koebele, 2021: 619–20). These groups may be characterised by:

- Institutional design, to ensure one ‘core negotiating group’ is supported by many ‘working groups’ conducive to informal ‘back and forth’ discussions and a boost in mutual trust following in person engagement ( 2021: 621).
- Wider politics, such as to ensure that enough credit is shared to reward participation. For example, high level organizations need the credit for setting up successful collaborative arrangements with good results, while participants need to detect their influence on the outputs of collaboration ( Koebele, 2021: 620; Koebele, 2020: 741).

There is value to less formal ‘soft spaces’, occupied by a smaller number of actors, grouped according to geography, sector, issue, or a specific task to be considered and fed back to the formal mechanism ( Arrona *et al.*, 2020: 127). For example, the main group could delegate responsibility for key tasks to a small working group, operating relatively informally, and recruiting external actors (such as research specialists or stakeholders) to provide information or be an audience for nascent ideas. These ad hoc groups are more flexible in terms of rules, and more conducive to innovation through open exchange and experimentation.

***Policy labs to encourage policymakers, researchers, stakeholders, and citizens to define problems and co-design solutions***

Policy labs aim to include policymakers, researchers, stakeholders, and/ or citizens in collaborative fora that share knowledge and promote ‘open, honest conversations around a policy topic’ ( Hinrichs-Krapels *et al.*, 2020: 1). Potential benefits include greater trust between participants, the translation of evidence and values into policy ideas, and the ability to identify ‘windows of opportunity’ to connect this activity to new policy agendas ( 2020: 2–3).

### Collective resources and skills: Ensure that participants know their roles and possess key skills

Effective collaboration involves people from different positions contributing different skills ( Ansell *et al.*, 2017: 481–2). A necessarily large and diverse set of skills includes the woefully underappreciated ‘soft skills’ connecting collaboration to ‘problem solving’, ‘communication’ and ‘creativity & innovation’ ( Saba *et al.*, 2021: 10), and the following role-specific skills which often require high levels of expertise.

***Process expertise, including how to deliver effective in-person collaboration***

Do policymakers have the willingness or ability to perform so many collaborative design and participation roles? If the answer is yes, identify the ‘personas’ of participants and relate them to an honest assessment of training needs ( Jacob *et al.*, 2021: 13; see also Bostaph and Wintrow, 2021 on essential collaborative skills). Archetypal personas may include actors with expertise in managing organisations, or spanning boundaries between organisations or professions, leading policy design and innovation, or harnessing scientific expertise, each with different training and support needs ( Jacob *et al.*, 2021: 11–12).

If the answer is no, ‘process experts’ should be on hand to guide them ( Molinengo *et al.*, 2021). Process expertise can describe ‘front stage’ skills in facilitating activities and social norms to foster knowledge exchange between participants in a respectful but challenging manner, and ‘back stage’ activities regarding who to involve and where and when to meet ( 2021: 4). Much of this work is essential but ‘invisible’ ( 2021: 10; Eneqvist & Karvonen, 2021: 186).

***External facilitators, to provide independent sources of expertise or conflict mediation***

External sources of activity may include ‘joint excursions, independent expert reports and comparative studies of solutions in other countries and jurisdictions that can challenge the taken-for-granted-knowledge and stimulate experimentation’ ( Ansell *et al.*, 2017: 481–2).

***Influential sponsors or champions within government departments***

Senior actors may need to be ‘sponsors and champions of collaborative policymaking’ to normalise the expectation that policy should be made in this way ( Ansell *et al.*, 2017: 481–2)

***Politicians with the means and motivation to connect collaborative work to political rules and agendas***

It is essential to understand how routine administrative collaboration connects to the macropolitical institutions that legitimise and influence this activity. In other words, ‘democratic political leadership is’ essential ‘to strengthen the dialogue between policymaking in collaborative governance arenas and institutions of representative democracy’ ( Sørensen *et al.*, 2020: 531). ‘Boundary spanning’ roles include to maintain relationships between many actors, connections between their aims, information-sharing and learning, and to spot ‘windows of opportunity that pave the way for a political alignment’ ( 2020: 535). One key aim is to establish the right mix of ‘hands off’ activity to establish the conditions for collaboration and foster participant autonomy, and ‘hands on’ activity to foster alignment between collaborative and political arenas ( 2020: 546–7). Another is to gauge the motivation of each politician to support collaboration across departments, which is not a given when departments have a clear rationale for operating separately and their leaders benefit from that arrangement ( Jacob *et al.*, 2021: 8; Klüser *et al.*, 2024: 532).

## Learning from comparable initiatives to foster more effective policymaking

While these insights emerge from studies of ‘collaborative policymaking’, very similar ideas have emerged from other approaches. Reports on the design principles and methods for ‘co-creation’ processes - between policymakers, stakeholders, and citizens - provide broad lessons for collaboration with colleagues across government. Dixon *et al.* (2021: 2–9) describe five steps:

1. Plan well. Do your homework on intended participants, use design methods to develop prototypes to focus collective attention, and establish good in-person and online means of communication, before initiating the more intensive process of co-production ( Dixon *et al.*, 2021: 7–9)
2. Attend to organisational design, which includes establishing the size of groups and right mix of participants, clarifying the governance structure for collective action including who or what provides the main authority, and considering a formal agreement that sets out collective expectations ( Dixon *et al.*, 2021: 7–9).
3. Foster open and creative discussion by setting up smaller working groups ( Dixon *et al.*, 2021: 7–9).
4. Use leadership to establish shared aims then facilitate and monitor progress ( Dixon *et al.*, 2021: 7–9).
5. Build trust through regular informal discussion (in person and online), backed by effective communication on progress, and perhaps independent facilitation ( Dixon *et al.*, 2021: 7–9).

Studies of ‘joined up government’ or ‘whole of government’ approaches examine attempts to improve policy coherence via more effective policymaking integration. For example, Carey *et al.* (2014: 9) distinguish between the (1) ‘Hard’ or ‘structural’ facilitators, including: a ‘mandate for change’ set by central government combined with sufficient control spread across multiple levels of government; clear ‘accountability and incentive mechanisms’; and sufficient resources, used flexibly in each relevant level of government, as well as the (2) ‘Soft’ or ‘cultural and institutional’ changes, including: a ‘strategic focus on collaboration’, ‘skill development’, a ‘rallying call’ for participants, and ‘information sharing’. Carey and Crammond’s (2015: 1022–8) review of ‘joined-up government’ research signals the need for:

- “a ‘supportive architecture’, where agreed aims are matched to the means to achieve them, with enough flexibility to adapt to the dynamics of coordination efforts
- mutually reinforcing changes at multiple levels of government, reinforced by shared targets
- high commitment by politicians, to cut through ‘administrative silos’ and ‘turf wars’
- strong ‘leadership’ to ensure that all relevant bodies sign up to changes
- skilful actors, in problem-solving, coordination, brokering agreements, and engaging with non-governmental actors
- leaders able to work inside and outside formal arrangements (while respecting the link between action and accountability)
- a manageable number of aims and policy instruments a powerful narrative to challenge business-as-usual approaches and give people a common purpose” (cited in Cairney *et al.*, 2022; see also Aoki *et al.*, 2024).

## Conclusion

This review synthesises academic and practitioner insights to offer pragmatic advice to policymakers seeking more effective *collaborative policymaking*. We relate this advice to a commonly told story of policy and policymaking complexity: policy problems require meaningful collaboration, by actors spread across multiple organisations and policy sectors. There are two main reference points for advice. Aspirational approaches relate advice to the pursuit of rationalist policy processes and the protection of democratic ideals. Policy theories explain why complex policymaking systems are not always conducive to collaboration. This context prompted a search for advice that offers hope for individual and collective action but tempered by policymaking reality. We summarise our interpretation of useful advice as follows.

### Collective Planning: Do your research to prepare for in-person meetings

Effective collaboration involves face-to-face discussion to boost trust, learning, and cooperation. However, most essential activities take place before meetings, including:

- Design and defend your rules for inclusion, which includes addressing trade-offs regarding group size and diversity.
- Signal respect for participants in the formal procedures for inclusion and collaboration. Anticipate the unintended consequences of innocuous informal rules and norms.
- Surface the values and beliefs of participants, to help set realistic expectations.
- Identify how to support the elements essential to mutual learning.
- Clarify and explain routines, including the mix of in-person and online collaboration.
- Identify the practices that can undermine effective collaboration, including poor planning and vaguely described activities.

### Collective identity: Create a clear sense of purpose for the collaboration

Effective collaboration is built on a collective sense of purpose, aided by these actions:

- Set a boundary around your work, establishing which issues and actions are relevant .
- Foster a common vision (guiding principle, strategy, objectives) and language.
- Define success for the collaboration as well as the policy, to inform measures of progress for the group as well as longer-term policy outputs and outcomes.

### Collective practices: Use creative methods to design a collaborative vision

Effective collaboration requires participants to articulate a shared vision, which can be facilitated by multiple methods including:

- Visualisation methods to map your policy problem, network, and intended response
- Design techniques to inform policy and reflect on collaboration

### Collective spaces: Create new forums to support collaboration

Formal and well-established policymaking rules and venues are essential, but they can be supported by new and more flexible spaces for collaboration, including:

- Working groups to support core groups, providing ‘soft spaces’ of governance to foster informal discussion and experimentation.
- Policy labs to encourage policymakers, researchers, stakeholders, and citizens to define problems and co-design solutions.

### Collective resources and skills: Ensure that participants know their roles and possess key skills

Effective collaboration requires the application of a large and diverse set of skills, many of which are role specific or require high levels of expertise, including:

- Process expertise, including how to deliver effective in-person collaboration.
- External facilitators, to provide independent sources of expertise or conflict mediation.
- Influential sponsors or champions within government departments.
- Politicians with the means and motivation to connect collaborative work to political rules and agendas.

While such advice may be generally useful, no studies provide a how-to guide. Rather, the in-context application of such principles may aid the initial design of collaborative policymaking, and prompt collective deliberation on how to proceed. Further, no review of this kind can do full justice to the pursuit of better policymaking, since studies of effective government are multi-faceted and require the combination of multiple reviews.

## Ethics and consent

Ethical approval and consent were not required.

## Data Availability

All data underlying the results are available as part of the article and no additional source data are required. Open Science Framework: Collaborative policymaking. https://osf.io/9bk6a/ This project contains the following extended data: - Structured bibliography Qualitative Systematic Review https://osf.io/ek8hd - Study Protocol https://osf.io/9zsa4 Open Science Framework: Collaborative Policymaking: a qualitative systematic review of advice for policymakers https://osf.io/5n6pa The project contains the following data PRISMA checklist https://osf.io/5n6pa Data are available under the terms of the Creative Commons Attribution 4.0 International license (CC-BY 4.0).
