# Supplementary material for: Collaborative Policymaking: a qualitative systematic review of advice for policymakers
Source: Open Res Eur. 2024 Sep 18;4:204. [Version 1] doi: 10.12688/openreseurope.18440.1 (PMC11568374; doi:10.12688/openreseurope.18440.1)
Supplement: Peer review history

# Reviewer report: Reviewer response for version 1

**DOI:** 10.21956/openreseurope.19928.r44776

**Published:** 2024-11-25

**Authors:** Yvonne La Grouw

**Affiliations:** 1 Utrecht University, Utrecht, The Netherlands

This article is a very welcome contribution to the literature and practical knowledge on collaborative policymaking. In this qualitative systematic review, the authors highlight the most important gaps in our knowledge on this topic: the need for collaboration does not automatically lead to collaborative action, and, design principles do not guarantee successful collaborative processes. These are very important issues in our current thinking about collaborative policymaking in both academia and practice – as the dominant narrative about collaborative policymaking is, indeed, aspirational. Next, the authors do give advice on how to deal with collaborative policymaking by identifying five main practices associated with effective collaboration. These broad principles could indeed help people and organisations to give direction to their collaborative endeavors. So, this is a thorough overview of the literature and I fully agree with the arguments made by the authors. I believe both academic and practitioners benefit from this review.

 I have some minor suggestions for improvement:

- The authors could be more explicit about how to deal with the challenges of collaborative policymaking *during *collaborative processes. Often, collaborative processes take a lot of time, and while design principles and inclusion practices were applied at the start of a collaboration, it does not guarantee that actors remain involved, engaged and motivated during a collaborative process. In fact, studies on collaborative processes often show how power dynamics create in- and exclusion during collaboration and collaborative inertia.
- Next, the role of power dynamics and dark sides of collaborative policymaking could get more attention in the paper. How do we deal with tokenistic collaboration? And to what extent can collaboration promise to really facilitate democratic ideals, or do power relationships, knowledge, skills and resources still matter during collaborations? What does this imply for practice? Do the more skilled and powerful actors always ‘win’ in collaborative processes? Or does collaboration, as an approach, avoid this?
- Regarding their advice to practice, the authors could consider to temper the expectations of collaborative policymaking: yes it is important and relevant, but also complex and messy. When people expect a messy process, it might help them to navigate the tensions of collaborative processes and accept the time and resources needed.
- In the methods section, there is a paragraph about the engagement with previous reviews. It would be interesting if the authors could elaborate on how the previous reviews inform this one.
- Also, the authors argue that they did not expect to find enough information to conduct a realist review, based on previous team experiences. Could the authors elaborate on these previous team experiences?
- Figure 2 is not sharp, maybe this could be improved. 

Are sufficient details of the methods and analysis provided to allow replication by others?

Yes

Are the rationale for, and objectives of, the Systematic Review clearly stated?

Yes

Is the statistical analysis and its interpretation appropriate?

Yes

Are the conclusions drawn adequately supported by the results presented in the review?

Yes

Reviewer Expertise:

collaborative decision-making, collaborative governance, preventive healthcare, health inequality

I confirm that I have read this submission and believe that I have an appropriate level of expertise to confirm that it is of an acceptable scientific standard.

# Reviewer report: Reviewer response for version 1

**DOI:** 10.21956/openreseurope.19928.r44768

**Published:** 2024-11-15

**Authors:** Bishoy Zaki

**Affiliations:** 1 Ghent University, Ghent, Belgium

This is a very well written article that tackles a very important issue, that is collaborative policymaking. The article makes several significant contributions, both to research and practice. The choice of methodological approach is highly appropriate, and the application is admirably robust.

 One of the most important contributions of this article is that it provides a grounded set of features on collaborative policymaking: planning and preparing to collaborate; creating a sense of collective purpose, such as by setting a boundary around the collaboration and co-producing a common vision; fostering creative methods to visualise collaboration and design policy; creating new forums to supplement formal collaboration; and clarifying the roles and skills essential to each collaborative task. Additionally, I find that the discussion of the general features of good collaborative policymaking design very important and warrants significant attention of practitioners.

 While the authors’ findings are certainly insightful, one particular finding is very telling, and should not go unnoticed, neither for practitioners nor for academics. The fact that the authors find much of the analysed literature does not often provide tangible and useable takeaways on how to better collaborate should be an important takeaway that guides future knowledge production in both communities, research and practice.

 I find the authors' reflection on the conceptual approaches to collaborative policymaking highly relevant and serves as an important basis for guiding the discussion. This becomes particularly important as the concept has always been somewhat fuzzy, and difficult to pin down. In terms of the analytical framework, the authors utilise a very comprehensive framework that touches on very important issues, often under-explored in literature (e.g., contexts and determinants of when, why, and how collaborative policymaking works, and equally important – the transferability of lessons distilled across contexts). Also, on the methodology, I salute the authors for breaking path with the field’s habit of using multiple scientific databases, in many cases, it is indeed not a very rewarding approach that introduces significant overheads, without commensurate returns.

 Of course, as in the ancient tradition of all academics doing reviews, reading a thought-provoking article stimulates some questions and curiosities.

- Perhaps one of the key areas I would have liked to read more about is the dark side of collaborative policymaking? Can it lead to adverse outcomes (in addition to situations where it is not done right?), are there situations where we should try to stay away from collaborative policymaking? If so, why? How? And what should we do?
- There are emerging views that argue that “collaborative governance” might not be the best way to address wickedness and complexity. Related to my previous point, perhaps this is also something the authors can address (Even if briefly).
- I believe the authors could tell us a bit more about their exclusion criteria. Also, maybe for those who are not familiar with Policy Commons, some more information there, and examples of how inclusion/exclusion was managed on this (in the main text).

I wish the authors all the best with this important project, and I look forward to reading more of their work on the topic.

Are sufficient details of the methods and analysis provided to allow replication by others?

Yes

Are the rationale for, and objectives of, the Systematic Review clearly stated?

Yes

Is the statistical analysis and its interpretation appropriate?

Yes

Are the conclusions drawn adequately supported by the results presented in the review?

Yes

Reviewer Expertise:

Policy process theory, policy learning, crisis policymaking, public governance

I confirm that I have read this submission and believe that I have an appropriate level of expertise to confirm that it is of an acceptable scientific standard.

# Reviewer report: Reviewer response for version 1

**DOI:** 10.21956/openreseurope.19928.r44774

**Published:** 2024-11-13

**Authors:** Daniel J Mallinson

**Affiliations:** 1 Penn State Harrisburg, Middletown, Pennsylvania, USA

This is an excellent piece of scholarship that raises important questions for both academics and practitioners. Cairney and Toomey set about to conduct a systematic literature review of the work on collaborative policymaking. The piece is full of insights that should make anyone studying collaborative policymaking/governance take stock. As their methodology makes clear, the boundaries of this loosely connected body of work makes such a review challenging. That is really the first insight, particularly given the fact that this piece aims to identifying practical lessons for governments from a literature that purports to study effective collaboration. The authors make a convincing case for their search, evaluation, and analysis approaches. Additionally, they are well experienced in this literature and the systematic review process. Thus, the methodology is sound, as well as honest about the limitations in marshalling a literature with hazy conceptual definitions and boundaries. The analysis is then broken into two main parts – collaboration in experience and in aspiration. As the authors note, the collective literature leans more towards the aspirational than the experiential. This does not mean that there are no empirical studies of collaboration, however it is fragmented. That said, the authors do offer very practical advice for governments based on their assessment of the literature. It runs the gamut, including the major topics of planning, collaboration identity, practice, creating collaborative spaces, and developing the roles and skills of collaborators. The authors admit that the application of the insights is context-dependent, but this is the most practical set of guidelines that I have yet read on collaborative policymaking/governance. While useful for practitioners, the piece should also be a strong nudge to researchers for more work on experience and less on aspiration. The aspiration is clear, but much needs to be done to identify how and when collaboration works, why it fails, and how scholarship can better inform practice.

Are sufficient details of the methods and analysis provided to allow replication by others?

Yes

Are the rationale for, and objectives of, the Systematic Review clearly stated?

Yes

Is the statistical analysis and its interpretation appropriate?

Yes

Are the conclusions drawn adequately supported by the results presented in the review?

Yes

Reviewer Expertise:

Policy process theory, cannabis policy, energy policy

I confirm that I have read this submission and believe that I have an appropriate level of expertise to confirm that it is of an acceptable scientific standard.
